# Performance of Automated Point-of-Care Respiratory Rate Counting versus Manual Counting in Children under Five Admitted with Severe Febrile Illness to Kisantu Hospital, DR Congo

**DOI:** 10.3390/diagnostics11112078

**Published:** 2021-11-10

**Authors:** Bieke Tack, Daniel Vita, Thomas Nsema Mbaki, Octavie Lunguya, Jaan Toelen, Jan Jacobs

**Affiliations:** 1 Department of Clinical Sciences, Institute of Tropical Medicine, 2000 Antwerp, Belgium; jjacobs@itg.be; 2 Department of Microbiology, Immunology and Transplantation, KU Leuven, 3000 Leuven, Belgium; 3 Hôpital Général de Référence Saint Luc de Kisantu, Kisantu, Democratic Republic of the Congo; dvitamayimona@gmail.com (D.V.); thomasnsema202020@gmail.com (T.N.M.); 4 Department of Microbiology, Institut National de Recherche Biomédicale, Kinshasa, Democratic Republic of the Congo; octmetila@yahoo.fr; 5 Department of Medical Biology, University Teaching Hospital of Kinshasa, Kinshasa, Democratic Republic of the Congo; 6 Department of Development and Regeneration, KU Leuven, 3000 Leuven, Belgium; jaan.toelen@kuleuven.be

**Keywords:** automated respiratory rate counting, fast breathing, point-of-care test, low resource setting, handheld pulse oximeter, severe febrile illness, bloodstream infection, severe malaria, WHO Integrated Management of Childhood Illness

## Abstract

To improve the early recognition of danger signs in children with severe febrile illness in low resource settings, WHO promotes automated respiratory rate (RR) counting, but its performance is unknown in this population. Therefore, we prospectively evaluated the field performance of automated point-of-care plethysmography-based RR counting in hospitalized children with severe febrile illness (<5 years) in DR Congo. A trained research nurse simultaneously counted the RR manually (comparative method) and automatically with the Masimo Rad G pulse oximeter. Valid paired RR measurements were obtained in 202 (83.1%) children, among whom 43.1% (87/202) had fast breathing according to WHO criteria based on manual counting. Automated counting frequently underestimated the RR (median difference of −1 breath/minute; p2.5–p97.5 limits of agreement: −34–6), particularly at higher RR. This resulted in a failure to detect fast breathing in 24.1% (21/87) of fast breathing children (positive percent agreement: 75.9%), which was not explained by clinical characteristics (*p* > 0.05). Children without fast breathing were mostly correctly classified (negative percent agreement: 98.3%). In conclusion, in the present setting the automated RR counter performed insufficiently to facilitate the early recognition of danger signs in children with severe febrile illness, given wide limits of agreement and a too low positive percent agreement.

## 1. Introduction

Globally, severe febrile illnesses, including malaria, pneumonia and sepsis, are leading causes of death in children under five years old [1,2,3]. To reduce under-five mortality, early treatment and/or referral of children with severe febrile illness is essential. However, the early detection of clinical danger signs indicating severe febrile illness remains a major hurdle to frontline health workers [3].

Fast breathing is a well-known clinical danger sign in children with pneumonia requiring antibiotics. In its Integrated Management of Childhood Illnesses (IMCI), the World Health Organization (WHO) therefore recommends to systematically measure the respiratory rate (RR) in children with breathing difficulties or cough [4]. Moreover, metabolic acidosis in severe malaria or bacterial sepsis can also cause fast breathing [5].

Despite its importance as a clinical danger sign, RR counting is frequently neglected or incorrectly performed by frontline health workers [3,6,7,8,9]. Manual RR counting can be difficult and time consuming, particularly in agitated children. Breathing cycles should be counted during one minute in a child at rest [10,11]. Furthermore, although timers and assisted counting methods exist, it is challenging to simultaneously focus on the child’s chest movements, count them, and time one minute [10].

Technology to automatically count RR has existed for many years but is not routinely used, and new technology continues to be developed [10,12]. Most automated RR counters are highly technological devices designed for continuous monitoring in high-resource settings [10,12]. In low- and middle-income countries, automated RR counting has not yet been adopted. Therefore, UNICEF launched the acute respiratory infection diagnostic aid (ARIDA) project and determined the target product profile of an automated RR counter [13]. Recently, two automated RR counters, i.e., a photoplethysmography-based (Masimo Rad G) [14,15,16] and accelerometer-based (Philips ChARM) [17,18,19,20] device have been evaluated in a low-resource setting. These studies revealed variable accuracy and some usability issues, such as slow performance and measurement failure [14,15,16,17,18,19,20]. However, for a full evaluation of the value of these two methods to detect fast breathing as a clinical danger sign, field studies should specifically address children with severe febrile illness in a low resource setting. Disease severity may influence accuracy [21] and usability and high sensitivity is most important in this population [3,10,22,23]. Finally, some recently developed devices are low cost and wearable, e.g., with a thermistor [24] or a strain gauge sensor [25], and might achieve high accuracy [12], but their usability and performance in severely febrile children and low resource or tropical settings should still be validated [3,10]. Therefore, field performance and usability data from currently available methods in this setting are also needed to allow comparison with more recently developed methods.

**Figure 1:**
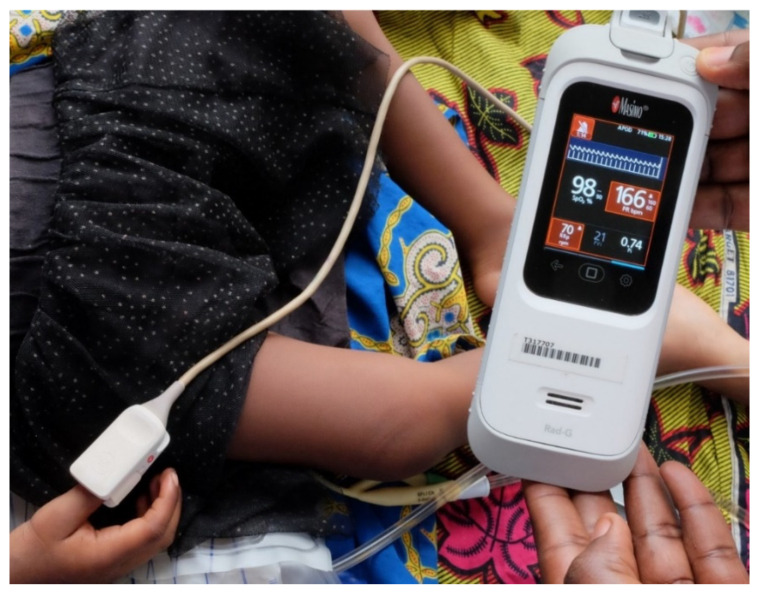
Automated respiratory rate counting with Masimo Rad G pulse oximeter. A one size fits all probe is attached to the thumb of a child admitted to Kisantu hospital with severe febrile illness. On the display, the plethysmogram is displayed on top, below the oxygen saturation of 98%, heart rate of 166/min and respiratory rate of 70/min are displayed. The heart rate and respiratory rate are too high and provoke a visual (red color) and sound alarm (silenced in the upper left corner).

With this study, we aimed to evaluate the performance of plethysmography-based respiratory rate counting by a handheld multimodal pulse oximeter (Figure 1; Rad G, Masimo, Irvine, CA, USA) in a population of hospital-admitted children with severe febrile illness in the Democratic Republic of the Congo (DR Congo). These field data will elucidate its value as a diagnostic tool to facilitate the early recognition of fast breathing as clinical danger sign in children with severe febrile illness in low-resource settings. 

## 2. Materials and Methods

The study was conducted at St. Luc Kisantu general referral hospital (Kisantu hospital) from 1 July until 31 August 2021 (dry season). Data were collected as part of the DeNTS study (NCT04473768), a prospective study on the clinical presentation of non-typhoidal *Salmonella* bloodstream infection in children aged >28 days and <5 years who are admitted with suspicion of a bloodstream infection. 

Kisantu hospital is the referral hospital of the Kisantu health district (locally referred to as health zone), a semi-rural area located in the Kongo Central province in the south-west of DR Congo. Kisantu hospital has a flat fee system for patients referred by first line health care centers from the Kisantu health district, which results in high bed occupancy rates in the hospital [26]. In Kongo Central, *Plasmodium falciparum* (*Pf)* malaria is holoendemic, i.e., 24% of children (6–59 months) had a positive blood microscopy test during a national health survey in 2013–2014 [27]. The same survey demonstrated that 69% of children (6—59 months) were anemic and 11% of children under five years old were suffering from acute malnutrition [27]. HIV-prevalence is relatively low (0.2% of the adult population) [27]. Since 2007, blood culture surveillance has been organized at Kisantu hospital by the National Institute for Biomedical Research (INRB, Kinshasa, Congo) and the Institute of Tropical Medicine (ITM, Antwerp, Belgium) [28,29,30,31]. Blood cultures are sampled and worked up free of charge as part of the routine clinical care [28,29,30,31]. Blood culture surveillance has demonstrated a high burden of non-typhoidal *Salmonella* bloodstream infections at Kisantu hospital, particularly in children under five years old in whom they cause up to 75% of culture-confirmed bloodstream infections [28,30,31].

Dedicated research nurses were trained to manually and automatically count the RR before study onset and were supervised by the principal investigator (B.T., pediatric resident) during the first six weeks of the DeNTS study. RR were counted upon inclusion in the DeNTS study, which was done as soon as possible after hospital admission (inclusions 7/7 days per week during working hours). A timer with an auditory alarm after 60 s was used for manual RR counting, based on visual observation of chest movements. Automated RR counting was performed with Masimo Rad G continuous pulse oximeter in the default APOD mode with the Masimo Rad G reusable finger clip sensor (Masimo, Irvine, CA, USA; Figure 1), according to the manufacturer’s instructions. The Masimo Rad G pulse oximeter is a multimodal device with a one-size-fits-all sensor that is placed on the tip of the thumb, finger or great toe, and that measures oxygen saturation, heart rate, respiratory rate, perfusion index and pleth variability index based on differential absorption of infrared light by oxygenated versus deoxygenated blood (spectrophotometry) and on pulse-related changes in the amount of arterial oxygenated blood in the tissues (photoplethysmography). The device determines the RR based on the cyclic variations in the photoplethysmogram and is designed to cover the range of 4–70 breaths per minute.

As recommended by the WHO, (i) children must be calm before and during RR counting and may not be eating or drinking and (ii) manual RR must be counted during 60 s [4]. In the study protocol, compliance with these conditions was mandated in addition to compliance to criteria adopted from previous studies on paired RR measurement with the Masimo Rad G pulse oximeter [32], i.e., (i) manual and automated RR had to be counted simultaneously, (ii) the Masimo Rad G pulse oximeter probe had to be correctly positioned and oriented and (iii) the Masimo Rad G had to display a definitive RR measurement. A definitive RR measurement was defined as a RR displayed in white, in contrast to preliminary RR measurements displayed in grey, when the Masimo Rad G was still internally confirming its RR count. Compliance with the WHO recommendations and the study protocol were self-reported by the research nurse in each child’s case report form. In the case of deviation from the WHO criteria or criteria for paired RR measurement in the study protocol, the paired RR measurement was considered as invalid for comparison and was excluded from further performance analysis. Fast breathing was defined according to WHO-criteria (Table 1). Upon inclusion in the DeNTS study, a trained research nurse also measured other clinical parameters and interpreted them according to the WHO-criteria; these are listed in Table 1. A trained study physician examined the child to detect signs of respiratory distress, i.e., grunting, nasal flaring or chest retractions. *Pf* malaria infections were diagnosed by malaria microscopy testing in the hospital laboratory. Bloodstream infections were diagnosed by growth of a pathogen from a blood culture, as described elsewhere [28,30,31].

Data were collected from a convenience sample of children included in the DeNTS study. A study duration of two months was chosen to obtain enough paired RR measurements to allow Bland-Altman analysis, which, as a rule of thumb, requires 100–200 paired observations [33]. Data analysis was performed in R with manual RR counting as a comparative method and a significance level of 0.05. To compare clinical characteristics of two groups, medians were compared with the Wilcoxon rank-sum test and proportions were compared with chi-square or Fisher exact test. The difference in median RR with manual versus automated RR counting was analyzed with a paired Wilcoxon-rank sum test. Correlation between manual and automated RR counting was assessed by calculation of the Pearson correlation coefficient, the Lins concordance correlation coefficient, which, in contrast to the Pearson correlation coefficient, also takes into account systematic bias, and the intraclass correlation coefficient type 3 according to Shrout and Fleiss to assess absolute agreement [34]. In addition, the root mean square error (RMSE), which measures systematic bias and random error, was calculated as √([∑(RR_automated_ − RR_manual_)^2^]/n). Due to non-normality of the data, a non-parametric variant of Bland-Altman analysis with calculation of median differences and 2.5th and 97.5th percentile limits of agreement was performed to assess the accuracy of the automated method. Manual counting per se is also subject to measurement variation and can, as a consequence, not be considered as a gold standard [11,20]. Therefore, categorical agreement, positive and negative percent agreement were calculated with their 95% confidence interval instead of sensitivity and specificity, respectively [11,35]. Percent agreements were compared between age groups with a proportion z-test. In addition, a kappa-statistic was calculated to assess the overall agreement between both methods. Finally, we tried to explain failure to detect fast breathing by the automated method by assessing the association between clinical signs and successful versus failed detection of fast breathing. This association was assessed by Fisher exact testing for categorical variables due to the small group sizes and by logistic regression for the continuous variable weight.

## 3. Results

### 3.1. Description of the Study Population

During the two month study period, 243 children were included in the DeNTS study, of which 49% (*n* = 119) were male. The included children had a median age of 18 months (p25–75: 10–29 months) and a median of three days of fever before hospital admission (p25–p75: 2–4 days). A third of the included children (82/243 (33.7%)) had received antibiotics to treat their current febrile illness prior to their hospital admission. Vital signs confirmed the severity of febrile illness of the population, with fever upon admission in 29.2% (*n* = 71), tachycardia in 65% (*n* = 158) and fast breathing according to manual RR counting in 44% (*n* = 107). Anemia was present in 65.4% (*n* = 159) of patients and 59.3% (*n* = 144) suffered from *Pf* malaria. In 22 (9.1%) children, microbial growth was detected in the blood culture. Non-typhoidal *Salmonella* accounted for 16 (72.7%) of them, and other pathogens were *Staphylococcus aureus* (*n* = 1), *Klebsiella species* (*n* = 3), *Pseudomonas species* (*n* = 1) and yeasts (*n* = 1). 

### 3.2. Description of Valid, Invalid and Unsuccesfull Paired RR Measurements

**Figure 2:**
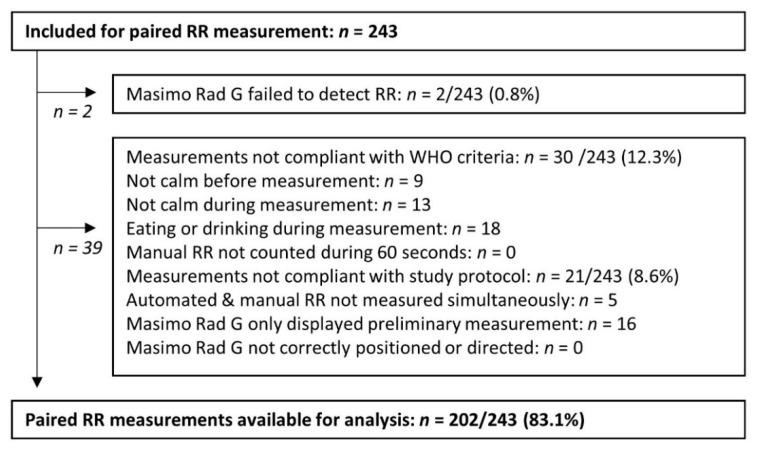
Overview of successful and valid paired respiratory rate (RR) measurement by manual counting and automated counting with the Masimo Rad G pulse oximeter.

Valid paired RR measurements were obtained from 202 (83.1%) of 243 included children. Automated RR counting was unsuccessful in two children. The first child was 24 months old and presented with hypoxia, respiratory distress, a relatively low heart rate of 80/min and severe dehydration. The child was diagnosed as having a severe *Pf* malaria infection and died upon the day of admission. The second child was 20 months old, presented with hypothermia of 34.2 °C and moderate acute malnutrition and suffered from a *Pf* malaria infection combined with a non-typhoidal *Salmonella* bloodstream infection. Paired RR measurements from 30 out of 243 (12.3%) included children were considered as invalid because the child was eating or drinking, or not calm before or during RR counting (Figure 2). Non-conformities to the study protocol were documented in 8.6% (*n* = 21) of included children (Figure 2). Registration of the preliminary RR accounted for three quarters (16/21 (76%)) of these non-conformities. This was frequently observed when, after a lengthy measurement trial, no definitive RR was displayed by the automated method. In addition, the research nurse reported slow (>2 min) performance of the automated method in 21% (*n* = 42) of valid paired RR measurements, from which 12 automated RR measurements required more than five minutes. 

Children with valid paired RR measurements had a median age of 19 months (p25–p75: 10–29 months). More than half of them were tachycardic and about a quarter of them were in respiratory distress (Table 2). Anemia was present in 129 (63.8%) children, among whom 12 had severe anemia. In 21.3% (*n* = 43) children, acute malnutrition was diagnosed, which was considered as severe acute malnutrition in 25 of them. More than half of the children suffered from *Pf* malaria infection and in 8.9% a bloodstream infection was confirmed (Table 2). The group of children with valid paired RR measurements did not significantly differ from the group with invalid paired RR measurements, except for a lower median weight in the latter group (Table 2). 

### 3.3. Automated Method Underestimated RR in Comparison to Manual Counting

**Figure 3:**
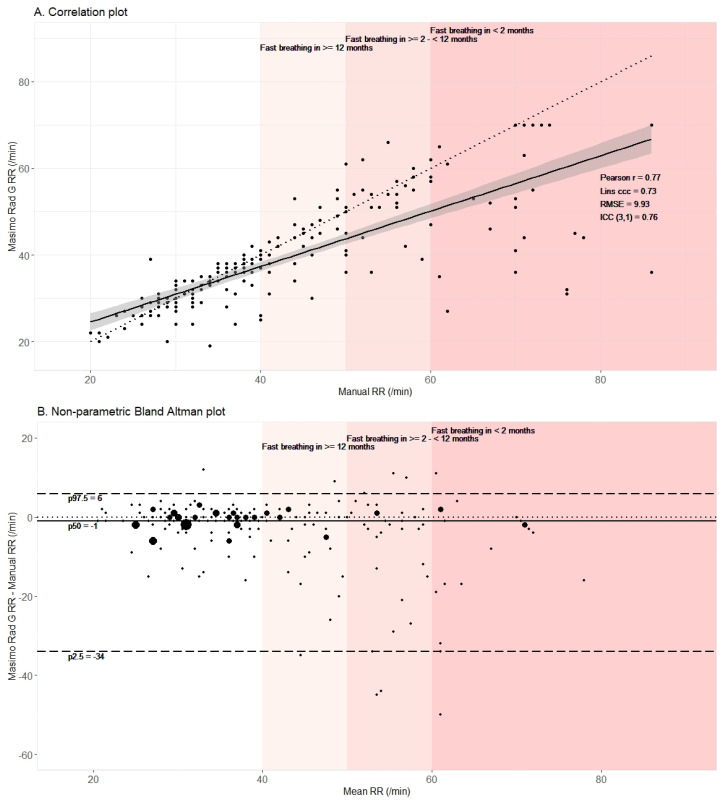
Correlation assessment (**A**) and Bland–Altman analysis (**B**) indicated insufficient accuracy of the automated respiratory rate (RR) counting by the Masimo Rad G pulse oximeter compared to manual RR counting. In the correlation plot (**A**), the regression line is drawn in black with its 95% confidence interval in grey. In the Bland–Altman plot (**B**), the median difference between automated and manual counting is indicated with a full black line and the 2.5th and 97.5th percentiles of this difference in dashed lines. In both plots (**A**) and (**B**), the dotted line displays the ideal situation and red colored zones indicate fast breathing according to age. Abbreviations: Pearson r: Pearson correlation coefficient; Lins ccc: Lins concordance coefficient; RMSE: Root mean square error; ICC (3,1): Intraclass coefficient type 3 according to Shrout & Fleiss; p: percentile.

The median RR by automated counting (36 breaths/min, Table 2) was significantly (*p* < 0.001) lower than the median RR by manual counting (39 breaths/min, Table 2). The maximum RR detected by the automated method was 70 breaths per minute, even in the case of higher RR by manual counting, which corresponded with the upper detection limit specified in the user manual. Although the automated and manually counted RR agreed relatively well for some children with a maximum difference of two breaths per minute in 56.4% (*n* = 114) of paired RR measurements, substantial underestimation by the automated method was observed in others, particularly in the case of fast breathing (Figure 3A). 

Bland–Altman analysis (Figure 3B) confirmed systematic underestimation of RR by the automated method with a median difference of −1 breath/minute (p25–p75: −4–1). The wide range between the 2.5th (−34 breaths/minute) and 97.5th (6 breaths/minute) limit of agreement demonstrated high variability in the differences between automated and manual RR counting. Bland–Altman analysis revealed no substantial differences in performance between children younger and older than 12 months (Figure S1). 

The Pearson correlation coefficient was 0.77 (95% CI: 0.71–0.82), confirming the linear association between the automated and manual method. Due to the systematic underestimation, the Lins concordance correlation coefficient was slightly lower, i.e., 0.73 (95% CI: 0.67–0.79). The type 3 intraclass correlation coefficient was 0.76 (95% CI: 0.69–0.81), which can be interpreted as moderate to good reliability [34]. The RMSE was 9.93 breaths per minute, which demonstrated an overall relatively large divergence of the automated method from manual RR counting. 

### 3.4. Automated Method Failed to Adequately Detect Fast Breathing as Defined by Manual Counting 

According to manual RR counting, fast breathing was present in 43.1% (*n* = 87) of 202 children with valid paired RR measurement. This proportion was significantly (*p* < 0.001) lower according to the automated method, which detected fast breathing in 33.7% (*n* = 68). Manual and automated RR counting resulted in the same classification in 88.6% (*n* = 179) of paired RR measurements. The automated method failed to detect fast breathing in 24.1% of the children with fast breathing according to manual counting, which corresponded with a positive percent agreement of 75.9% (Table 3). Positive percent agreement was lower in children less than one year old (65.6%, 95% CI: 46.8–80.8%), when compared to children between one and five years old (81.8%, 95% CI: 68.6–90.5%), albeit not statistically significant (*p* = 0.15). Children in whom the automated method failed to detect fast breathing were not only slightly younger, but also had a lower median weight, were more frequently severely malnourished and were less frequently infected with *Pf* malaria, albeit again not statistically significant (Table 4). Detection of fast breathing by the automated method but not by manual counting was rare (*n* = 2) and negative percent agreement was high (98.3%; Table 3). Overall categorical agreement, according to the kappa-statistic of 0.76, was substantial (Table 3).

## 4. Discussion

The present study described the field performance of the Masimo Rad G pulse oximeter for automated RR counting in comparison to manual RR counting in hospital-admitted children with severe febrile illness. When compared to the target product profile for automated RR counting developed by UNICEF, the automated method performed insufficiently in this study population: in almost half of the paired RR measurements, the prespecified accuracy of a maximum difference of two breaths per minute with the manual comparative method was not met [13]. Moreover, limits of agreement were wide, which points to high variability. The automated method frequently underestimated the RR, particularly in the group of interest, i.e., children with fast breathing. As a result, the automated method failed to detect fast breathing in one out of four children with fast breathing according to manual RR counting. Although user-friendliness was not formally assessed in this study, slow performance in one out of five children, sometimes in the absence of a definitive RR measurement after several minutes, limits its applicability in the field in this context. 

Previous studies in children under five years old in low-resource settings also reported difficulties to perform RR counting according to the WHO guidelines and a suboptimal performance of Masimo Rad G pulse oximeter for automated RR counting. A previous hospital-based study in Ethiopia from the ARIDA group assessed the usability of the Masimo Rad G pulse oximeter and reported that children were feeding or drinking, or not calm before or during RR counting, in up to 15% RR measurements, which corresponds with our observations [14]. They also reported slow performance with a median time of almost 6 min necessary for automated RR counting [14]. Finally, they reported failure to count the RR with the automated method after three attempts in 8% of children [14]. When compared to our study, an Indian study in children under five that presented at the outpatient or emergency department reported better performance of Masimo Rad G, with a lower and less variable underestimation (mean difference −0.4, 95% CI limits of agreement −7–6; comparative method: manual RR counting) [15]. In contrast to the Indian study and the present study, a study in Nigerian children under five years old hospitalized with severe acute malnutrition reported a slight overestimation of the RR with the Masimo Rad G pulse oximeter (mean difference +1.3 breaths per minute, comparative method: manual RR counting) [16]. The differences in observed performance between the studies might be caused by differences in the clinical characteristics of the included children, which, unfortunately, were largely unreported in these studies. The Masimo Rad G pulse oximeter relies on breathing- and pulse-related changes in the amount of arterial oxygenated blood in the tip of the finger or toe. Decreased tissue perfusion and anemia can impair the signal quality and can result in unsuccessful or inaccurate measurements [21]. In younger children and respiratory distress, breathing may be irregular and intermittent, which may further complicate the calculation of RR based on the photoplethysmogram [10]. In the present study, a relatively high proportion of children were younger than one year old, were tachycardic, were in respiratory distress, were anemic or were malnourished. The severity of febrile illness of the study population may explain the poorer performance of the automated method in our study when compared to the previous two studies, but may also more accurately reflect the clinical profile of children with fast breathing who should urgently be referred or treated [36,37]. Performance and user-friendliness of the Masimo Rad G pulse oximeter in this population may benefit from improved engineering to resolve the underestimation of fast breathing rates, flattening all RR above 70 breaths per minute and slow performance.

This study has some limitations. Firstly, there is no gold standard for RR counting [11] and manual RR counting is also subject to interobserver variability, particularly at faster breathing rates [38,39]. However, in the present study, dedicated research nurses were trained before data collection and minimal variation was observed between RR that were manually counted by a research nurse and a pediatric resident in the first six weeks after training. Secondly, the Masimo Rad G continuous pulse oximeter in APOD mode might have been less sensitive than the Masimo Rad G pulse oximeter that was specifically designed for spot checks. Thirdly, the anatomical site of automated RR measurement, i.e., finger, thumb or great toe, was not registered in this study. Previous studies demonstrated body site-specific differences in the accuracy of photoplethysmography-based RR measurement [40], amongst others due to differences in the waveform [41]. However, the Masimo Rad G user manual recommends all three sites for measurements in children and infants, with a preference for the great toe or thumb in children below 10 kg. In addition, data were collected during the dry season in a single hospital and findings may not be generalizable to other seasons or settings, e.g., to frontline health care centers. However, the hospital setting allowed us to obtain data from children with severe febrile illness, which is the population of largest interest for screening of danger signs. Such data included the presence of anemia, *Pf* malaria infection and blood culture confirmed bloodstream infection. In the rainy season, we would expect even higher proportions of children with *Pf* malaria, anemia, non-typhoidal *Salmonella* bloodstream infections and malnutrition, which might result in increased disease severity and further impair the performance of automated RR counting. Furthermore, research nurses were not blinded and the compliance to WHO recommendations and the study protocol was self-reported, which is a method that may be prone to reporting bias. Finally, sample size of the group of children with fast breathing may have been too small to reach statistical significance for associations with the clinical characteristics that may cause failure to detect fast breathing by the automated method. 

Due to its insufficient performance, we do not recommend the use of a Masimo Rad G pulse oximeter in its present version for automated RR counting in children under five years old with severe febrile illness. WHO currently recommends the use of an acute respiratory infection timer (ARI) from health post to the district hospital level [42]. This small device is started by pressing its center and LED lights indicate that it is counting and it beeps after 60 s [11]. In a multicenter study in hospital-admitted children under five years old in low-resource settings from the ARIDA group, manual RR counting with the ARI was compared to automated capnographic RR counting [11]. A maximum difference of two breaths per minute was observed in 32% of paired measurements. Although this is lower than the 56.4% agreement in the present study, overall, the ARI was more precise, with substantially tighter limits of agreement (mean difference = −0.6 breaths/min, 95% CI limits of agreement: −2.5–1.3). Nevertheless, as in the present study, the ARI underestimated the RR more in children with fast breathing, which translated to a positive percent agreement of only 54%. The use of counting beads, which assist the health care worker to count and which indicate fast breathing by colored beads, did not improve detection of fast breathing [11]. Therefore, other methods to assist or automate RR counting are urgently needed. In the above mentioned multicenter ARIDA study, phone applications (Rrate and Respirometer) based on a tap for each breath, did not perform better than manual RR counting with the ARI [11]. To our knowledge, the only other automated RR counter that was assessed in low-resource settings is the Philips Children’s Respiratory Monitor (ChARM; Philips India Limited, Calcutta, India), which is a small accelerometric device that is applied to the child’s chest and counts the chest’s movements. An agreement study was organized by the ARIDA group in hospitalized Ethiopian children under five years old, but was stopped early due to a device programming fault [20]. Performance of automated RR counting with ChARM was similar to the one of the Masimo Rad G pulse oximeter, with a mean difference of −1.1 breath per minute, wide 95% CI limits of agreement (−19.6–17.4 breaths/min), a kappa-statistic of 0.65 and a positive percent agreement of 81.5% [20]. Moreover, usability studies have shown that it also performed slowly with a mean performance time of more than three minutes [17,19] and the important limitation that breathing rates often increased after its application around the child’s chest due to agitation [18]. 

In the absence of accurate point-of-care devices for automated RR counting, fast breathing will remain an underdiagnosed danger sign. Systematic screening for danger signs by frontline health workers is an essential step to overcome the last mile gap and ensure that poor and rural communities also have access to the care cascade, i.e., screening, diagnosis, treatment and treatment completion and control [23]. However, frontline health workers do not possess the time, focus and experience to reliably count the RR or to simply count the RR at all [3,6,7,8,9]. As a result, screening of febrile illness in children is incomplete, fast breathing is overlooked and children with severe febrile illness requiring urgent referral or treatment are missed [3,23]. To increase compliance with the WHO IMCI guidelines to count the RR, the search for a reliable and user-friendly point of care device should be continued and based on the REASSURED criteria, i.e., real-time connectivity, ease of specimen collection, affordability, sensitivity, specificity, user-friendliness, rapid and robust, equipment-free or simple, and deliverable to end users [22,23]. The present version of Masimo Rad G pulse oximeter may benefit from improved engineering. Moreover, in high-resource settings, other RR measurement methods based on the exhaled breath, thoracic effort, respiratory sounds or indirect effects on cardiovascular physiology exist [10]. These methods should be further developed for implementation in tropicalized point-of-care devices. Their performance in terms of accuracy and reliable detection of fast breathing, usability and acceptability should be tested with field studies in low resource settings at frontline and hospital levels. 

## Figures and Tables

**Table 1:** Measurement and interpretation of clinical signs according to WHO [4,5].

Clinical Sign	Diagnostic Device/Technique	Definition
**Respiratory rate**	Manual or Automated RR with Rad G continuous pulse oximeter *(Masimo, Irvine, CA, USA)*	**Fast breathing**: <2 months: ≥60 breaths/min ≥2−<12 months: ≥50 breaths/min ≥12 months: ≥40 breaths/min
**Heart rate**	Rad G continuous pulse oximeter *(Masimo, Irvine, CA, USA)*	**Tachycardia**: <12 months: >160/min ≥12 months: >120/min
**O_2_ saturation**	Rad G continuous pulse oximeter *(Masimo, Irvine, CA, USA)*	**Hypoxia:** <90%
**Tympanic temperature**	Genius 3 *(Covidien, Mansfield, USA)*	**Fever:** >37.5 °C **Hypothermia:** <35.5 °C
**Capillary hemoglobin**	Hemocue 801 *(Hemocue, Angelholm, Sweden)*	**Severe anemia:** <5 g/dL **Moderate anemia:** ≥5−≤ 9.3 g/dL
**Malnutrition**	Weight: seca 876 scale *(seca, Hamburg, Germany)*	**Severe acute malnutrition:** - weight for height <−3 SD - peripheral upper arm circumference <115 mm if ≥6 months - or bilateral edema **Moderate acute malnutrition:** - weight for height ≥−3–<−2 SD - or peripheral upper arm circumference ≥115 mm−<125 mm if ≥6 months

**Table 2:** Clinical characteristics of the children from whom valid paired respiratory rate (RR) measurements were available compared with children in whom paired RR measurements were invalid due to non-compliance with WHO-criteria for RR counting or the study protocol. Medians were compared with the Wilcoxon signed rank test. Proportions were compared with the chi-square test or with the Fisher exact test if specified as such.

	Valid RR Measurements: *n* = 202	Invalid RR Measurements: *n* = 39 i.e., Non-Compliance with WHO- Criteria or Study Protocol	
**Paired RR measurement**	**Median (p25–p75) or *n* (%)**		***p*** **-value**
Manual RR counting (/min)	39 (32–52)	40 (34–52)	0.32
*Fast breathing (according to WHO)*	87 (43.1%)	19 (48.7%)	0.64
Automated RR counting (/min)	36 (32–46)	37 (34–46)	0.31
*Fast breathing (according to WHO)*	68 (33.7%)	12 (30.8%)	0.87
**Clinical characteristics**	**Median (p25–p75) or *n* (%)**		***p*** **-value**
Age			0.14 ^Fisher^
*<2 months*	1 (0.5%)	1 (2.6%)	
*≥* *2–<12 months*	60 (29.7%)	15 (38.5%)	
*≥* *12 months*	141 (69.8%)	23 (59.0%)	
Male gender	104 (51.5%)	15 (38.5%)	0.19
Weight (kg)	9.8 (8.1–11.9)	8.3 (6.8–10.9)	**0.03**
Tympanic temperature			0.56 ^Fisher^
*Fever (>37.5 °C)*	57 (28.2%)	14 (35.9%)	
*Hypothermia (<35.5 °C)*	4 (2.0%)	0	
Tachycardia (according to WHO criteria)	130 (64.4%)	28 (71.8%)	0.48
Hypoxia (< 90%)	8 (4.0%)	1 (2.6%)	1 ^Fisher^
Respiratory distress (grunting/nasal flaring/chest retractions)	47 (23.3%)	8 (20.5%)	0.87
Anemia			0.45 ^Fisher^
*Severe anemia (<5 g/dL)*	12 (5.9%)	2 (5.1%)	
*Moderate anemia (* *≥* *5–* *≤* *9.3 g/dL)*	117 (57.9%)	27 (69.2%)	
Malnutrition (according to WHO)			0.23 ^Fisher^
*Severe acute malnutrition*	25 (12.4%)	8 (20.5%)	
*Moderate acute malnutrition*	18 (8.9%)	5 (12.8%)	
Malaria infection (positive microscopy)	121 (59.9%)	21 (53.8%)	0.60
Bloodstream infection (blood culture confirmed)	18 (8.9%)	3 (7.7%)	1 ^Fisher^

**Table 3:** Categorical agreement of fast breathing detected by manual counting (comparative method) versus automated counting with Masimo Rad G pulse oximeter. Percentages between brackets represent the fraction from all paired RR measurements available for analysis (*n* = 202). Abbreviations: PPA: positive percent agreement; NPA: negative percent agreement; Kappa: kappa-statistic.

	Fast Breathing by Manual Counting	No Fast Breathing by Manual Counting	Total
**Fast breathing by automated counting**	66	2	68 (33.7%)
**No fast breathing by automated counting**	21	113	134 (66.3%)
**Total**	87 (43.1%)	115 (56.9%)	202
**Agreement** **(95% CI)**	PPA: 75.9% (65.3–84.1%)	NPA: 98.3% (93.2–99.7%)	Kappa: 0.76 (0.67–0.85)

**Table 4:** Comparison of clinical characteristics of children in whom the automated method failed to detect fast breathing. Fast breathing was defined according to the age-related WHO-criteria and based on the manually counted respiratory rate. Weight was analyzed as a continuous variable, i.e., presented as median with 25th and 75th percentile and calculation of Odds ratio and *p*-values by logistic regression. All other variables were categorical and therefore presented as numbers and group percentage. Their odds ratios and *p*-values were calculated by Fisher exact testing.

Performance of the Automated Method in Comparison to Manual Counting	Fast Breathing Detected *n* = 66	Failure to Detect Fast Breathing *n* = 21	Odds Ratio Failure	
**Clinical Characteristics**	***n* (%) or Median (p25–p75)**		**OR (95% CI)**	***p*-Value**
Infant (reference: ≥12 months)			2.33 (0.77–7.23)	0.12
*<2 months*	*0*	*1 (4.8%)*		
*≥2*−*<12 months*	*21 (31.8%)*	*10 (47.6%)*		
Weight (kg)	10 (8–12)	9 (6–11)	0.89 (0.74–1.07)	0.21
Fever (>37.5 °C)	31 (47.0%)	6 (28.6%)	2.19 ( 0.70–7.79)	0.21
Respiratory distress (grunting/nasal flaring/chest retractions)	29 (43.9%)	7 (33.3%)	0.64 (0.19–1.97)	0.45
Anemia (reference group: no anemia)			0.66 (0.21–2.15)	0.43
*Severe anemia* *(<5 g/dL)*	*7 (10.6%)*	*0*		
*Moderate anemia* *(≥5*−*≤9.3 g/dL)*	*40 (60.6%)*	*13 (61.9%)*		
Malnutrition (reference: no acute malnutrition)				
*Severe acute malnutrition* *(according to WHO)*	*9 (13.6%)*	*6 (28.6)*	*2.40 (0.60–9.16)*	*0.19*
*Moderate acute malnutrition* *(according to WHO)*	*6 (9.1%)*	*1 (4.8%)*	*0.61 (0.01–5.73)*	*1.0*
Malaria infection (positive microscopy)	51 (77.3%)	12 (57.1%)	0.40 (0.12–1.28)	0.09
Bloodstream infection (blood culture confirmed)	6 (9.1%)	2 (9.5%)	0.95 (0.15 –10.40)	1.0

## Data Availability

The data presented in this study are available on request from the corresponding author. The data are not publicly available due to an embargo on the data until the data collection, data analysis and manuscript publication of the DeNTS study are finalized.

## Supplementary Materials

The following are available online at https://www.mdpi.com/article/10.3390/diagnostics11112078/s1, Figure S1: Bland-Altman analysis stratified per age group.
