# Supplementary material for: Performance of Automated Point-of-Care Respiratory Rate Counting versus Manual Counting in Children under Five Admitted with Severe Febrile Illness to Kisantu Hospital, DR Congo
Source: Diagnostics (Basel). 2021 Nov 10;11(11):2078. doi: 10.3390/diagnostics11112078 (PMC8623579; doi:10.3390/diagnostics11112078)

Performance of Automated Point-of-Care Respiratory Rate Counting versus Manual Counting in Children under Five Admitted with Severe Febrile Illness to Kisantu Hospital, DR Congo

Bieke Tack, Daniel Vita, Thomas Nsema Mbaki, Octavie Lunguya , Jaan Toelen , Jan Jacobs
Correspondence: btack@itg.be

Figure S1. Bland-Altman analysis stratified per age group indicated insufficient accuracy of the automated respiratory rate (RR) counting by the Masimo Rad G pulse oximeter compared to manual RR counting, both in children below 12 months (A) and between 12 and 59 months old (B). The median difference between automated and manual counting is indicated with a full black line and the 2.5th and 97.5th percentiles of this difference in dashed lines. The dotted line displays the ideal situation and red colored zones indicate fast breathing according to age.

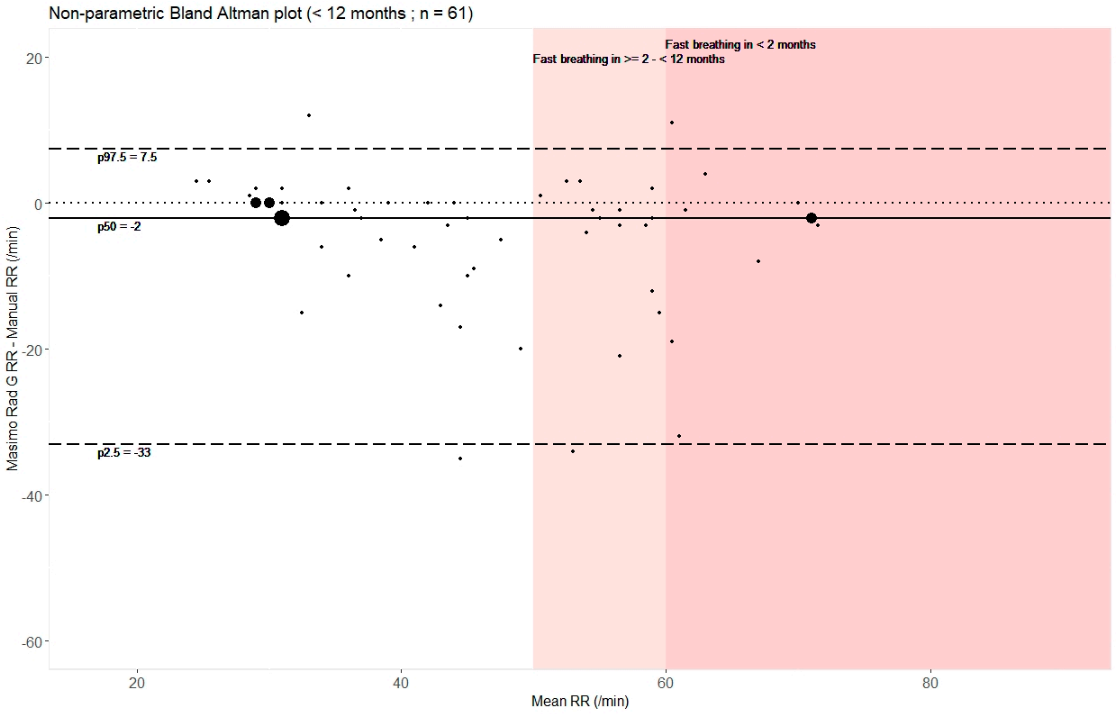

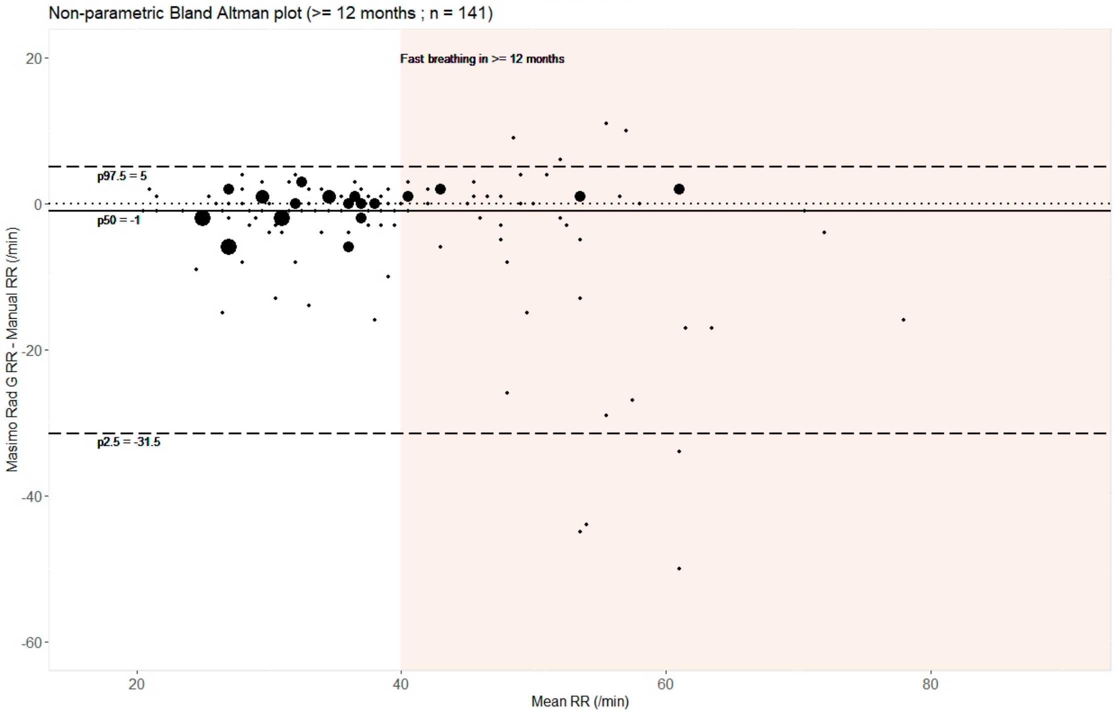

Supplement: Supplementary file 1 [file diagnostics-11-02078-s001.zip › diagnostics-1443935-supplementary.pdf]
